# Patient reported outcome measures (PROMS) for body image in dermatology: A systematic review

**DOI:** 10.1002/ski2.167

**Published:** 2022-09-14

**Authors:** Johannes Kjeldstrup Kristensen, Corina Nielsen, Nora Haloob

**Affiliations:** ^1^ Hud og Hyperhidrose‐klinikken Copenhagen Denmark; ^2^ Imperial College Healthcare NHS Trust London UK

## Abstract

**Introduction:**

It is widely acknowledged that negative body image perception is linked to anxiety, depression, and body dysmorphic disorder. However, there is no gold standard, body image related patient reported outcome measure in use, specific for dermatologic disease, despite evidence to suggest a high prevalence of mental health problems relating to body image in this group of patients.

**Aim:**

The aim of this study was to perform a review of body image Patient Reported Outcome Measures (PROMs) used in dermatology and to evaluate their effectiveness.

**Methods:**

Searches were performed in the major databases. Two investigators independently performed full text evaluation by applying an established checklist to evaluate the conceptual model, content validity, reliability, construct validity, scoring and interpretability and respondent burden.

**Results:**

Six different PROMs were identified of which only one was fully validated. There was a significant lack of patient involvement in the development of PROMs in this context.

**Conclusions:**

We therefore encourage further research in this field to improve the quality of evidence to better understand the relationship between mental health and dermatologic disease.

## Graphical abstract

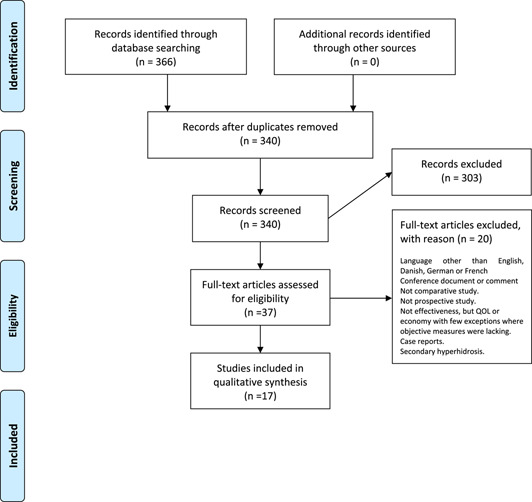

SIGNIFICANCE: Many dermatological conditions can have a detrimental impact on patients' perception of their body image and can contribute to mental health problems such as anxiety, depression, and body dysmorphic disorder. A systematic review was performed and identified six PROMs within this context. Unfortunately, only one of these were fully validated while the remaining had significant shortcomings in particular with regard to a lack of patient involvement during development. This calls for more work to do/be done to help researchers and clinicians evaluate the mental health effects dermatologic conditions may have on this patient cohort.

1

**What is already known about this topic?**

- A Review or Systematic Review has not been published previously.

**What does this study add?**

- This study lists the patient reported outcome measures (PROMs) used in dermatology to measure body image. This is important as a deranged body image can lead to Body Dysmorhic Dysorder. Not all PROMs are validated properly and patient participation during construction was not always the case. This could be unethical and should be corrected in future work on these PROMs.

Body image is a multidimensional construct that can be defined as the subjective emotions surrounding the degree of satisfaction an individual has with their appearance.^1^ An Individuals level of concern regarding their body image can be quantified on the Body Image Concern (BIC) scale and has been shown to strongly correlate with quality‐of‐life scores.^2^, ^3^ Furthermore, high BIC scores have been linked to psychological disorders including Body Dysmorphic Disorder (BDD).^4^ BDD which was previously classified as a somatoform disorder, has now been defined within obsessive‐compulsive disorders in Diagnostic and Statistical Manual of Mental Disorders (DSM‐5). A relationship to major depression, Obsessive Compulsive Disorder (OCD) and Social Phobia has been demonstrated.^5^ The prevalence of BDD in patients with dermatological conditions is reported as ranging between 4.9% and 36% compared with just 1.8–2.3 of the general population.^6^ In addition, a negative body image has been linked to anxiety and depression in dermatology patients.^7^

To date, no study has assessed which body image scoring tool is most reliable and fit for purpose and currently there are a variety of tools being adopted in the study of this topic which has resulted in a significant heterogeneity in data interpretation.

We set out to evaluate which body image Patient Reported Outcome Measure (PROM), relating to dermatological disease, reported within the published literature over the last 10 years, have undergone a validation process, as well as a critical appraisal.

This paper will highlight the current strengths, weaknesses and shortcomings in PROMs used to measure body image in dermatology patients which may inform future study design and clinical mental health evaluation of this high‐risk group.

## 2 METHODS

The methods for this systematic review were developed according to the recommendations from the Preferred Reporting Items for Systematic Reviews and Meta Analyses Protocol (PRISMA‐P) statement.^8^ The protocol for the study has been registered in The International Register of Systematic Reviews (PROSPERO): CRD 42021240444.

We planned to search several databases. We concentrated on generic (or if possible) specific instruments measuring body image in dermatologic diseases. The second aim was to perform a quality appraisal of all the discovered instruments. To avoid a subjective and to obtain an objective evaluation we chose to use the COSMIN approach (The Consensus based Standards for the selection of health Measurement Instruments)^9^ as modified by Francis.^10^ The modified version reduces the number of items from 119 to 17.

**FIGURE 1:**
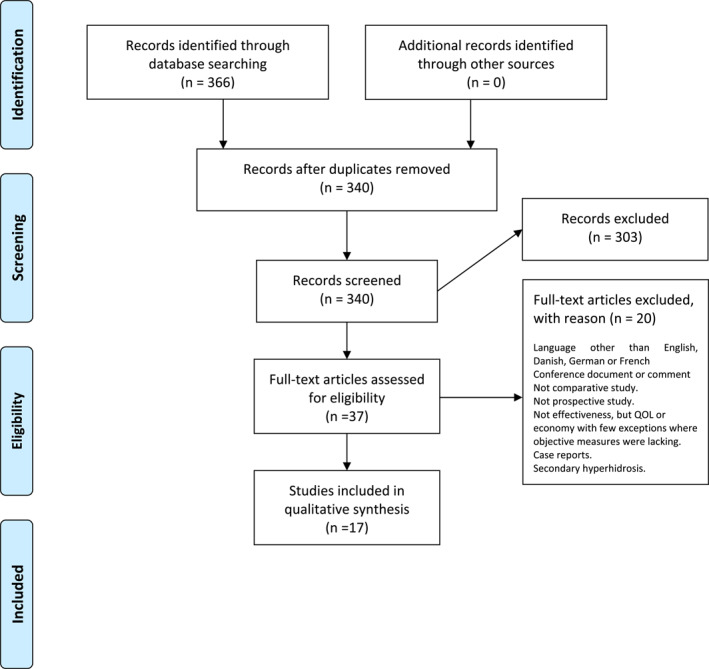
Flow chart of systematic literature search

In study part one, a systematic literature search was performed in PubMed, **EMBASE, Scopus, PSYCH Info and Cochrane Library databases using Medical Subject** Headings (Mesh) ‘body image’, combined with ‘skin diseases’, ‘dermatology patients’, ‘PROMS’, ‘surveys’ and ‘questionnaires’. We chose studies that had been published between 2010 and present and stratified the results according to the PRISMA flow chart (Figure 1). Only PROMs published more than once in the literature were included in this study.

In Study part two, a second search was performed for each PROM to identify articles pertaining to evaluation of the measure. Articles with paediatric data were not evaluated. We performed hand searches as well in a few published surveys of generic instrument concentrating on other subjects, as no previous review of body image in dermatology was found except for a book chapter.^11^

Evaluation of **psychometric properties** provide a level of evidence that an instrument is fit for the purpose.

**Reliability** assesses the extent to which a PROM tool yields consistent and reproducible results. The most important test for reliability is (1) test‐retest measures. (2) Internal consistency (or reliability) is often measured using Cronbach's alfa. As this measure is influenced by the number of items, users of this test have to be aware of questionnaires with redundant questions. Alfa should be not lower than 70 and not higher than 90.^12^

**Validity** describes the extent to which an instrument measures what it purports to measure. This is specific to the population and setting. (1) Construct validity signifies that the items provide distinctive clinical information. This is often explored using factor analysis and correlation coefficients. ‘Face validity’ is often used instead. (2) Criterion validity: This can be described as (a) predictive validity which means ability to predict response to treatment and clinical outcome and (b) concurrent validity where the correlation with another previously validated instrument is explored.

**Sensitivity** means that the items should be able to discriminate between different groups of patients, between patients and controls, it should be able to give meaningful results in clinical trials, be able to measure wanted or unwanted effects, measure active versus placebo. Sensitivity is crucial when treatment effects are small.

**Sensibility** (or clinical utility). The instrument should be easy to use, be short, use the right wording, be calibrated, and facilitate patient‐clinician interaction.

As previously mentioned, we adopted a modified COSMIN approach.^9^, ^10^

As the COSMIN approach was found to be too complex to use without modifications. Instead, the simplified version incorporates the critical features highlighted in COSMIN and other relevant literature^13^, ^14^ and hence will enable even unexperienced researchers to appraise a wide variety of PROMs.^10^

The quality of the individual papers can be judged indirectly from the scoring list (Table 2).

## 3 RESULTS

Table 1 shows the items in the short form PROM appraisal.^10^

**TABLE 1:** Simplified approach

Conceptual model Has the PRO construct to be measured been specifically defined? Has the intended respondent population been described? Does the conceptual model address whether a single scale ore multiple subscales are expected?
Content validity 4. Is the evidence that members of the respondent population were involved in the development of the PRO measure? 5. Is there evidence that content experts were involved in the development of the PRO measure? 6. Is there a description of the methodology by which items/questions were derived?
Reliability 7. Is there evidence that the reliability of the PRO measure was tested (e.g.: test‐retest, internal consistency)? 8. Are reported indices of reliability adequate?
Construct validity 9. Is there reported mathematical justification that a single scale or multiple subscales exist in the PRO measure (e.g.: factor analysis, item response theory). 10. Is the PRO measure intended to measure change over time? If yes, is there evidence of both test‐retest reliability and responsiveness change? Otherwise there may be an explicit statement that this PRO measure, is not intended to measure change over time. 11. Are there findings supporting expected correlations with existing PRO measures or other clinical data? 12. Are there findings supporting expected differences in scores between known groups
Scoring and interpretation 13. Is there documentation how to score the PRO measure? 14. Has a plan for managing and/or interpreting missing responses been described? 15. Is there information on how to interpret the PRO measure scores?
Respondent burden and presentation 16. Is time to complete reported and reasonable? If not, are number of questions appropriate for the intended application 17. Is the entire PRO measure available for public viewing?

The literature search is summarized in the PRISMA diagram (Figure 1).

We found six PROMS published in 17 papers and validated in 10 papers. One PROM was specific to dermatology, while the other five were generic.

The main properties of each PROM were extracted, and the results are presented in the text.

Their psychometric properties are presented in Table 2. From this table it is possible to deduct the quality of the papers produced with each individual PROM.

**TABLE 2:** Level of evidence per measurement property and PRO

	CBIS^a^	CBIS^a^	BIQLI^a^	BIQLI^a^	BIS^a^	BIS^a^	ASI‐R^a^	BISS^a^	BISS^a^	MBSRQ^a^
		Japanese		Danish		Portuguese			Spanish	
Measurement property										
Coceptual Model										
Has the PRO construct to be measured been specifically defined?	1	1	1	1	1	1	1	1	1	1
Has the intended respondent population been described?	1	1	1	1	1	1	1	1	1	1
Does the conceptual model address whether a single scale or multiple subscales are expected?	0	0	1	1	1	1	1	0	1	1
Content validity										
Is there evidence that content experts were involved in the development of the PRO measures?	0	0	0	1	1	1	0	0	1	0
Is the evidence that members of the respondent population were involved in the development of the PRO measures?	1	1	1	1	1	1	1	1	1	1
Is there a description of the methodology by which items/questions were derived?	0	0	0	1	1	1	1	0	0	0
Reliability										
Is there evidence that the reliability of the PRO measure was tested (e.g.: test‐retest, internal consistency)?	1	1	1	1	1	1	1	1	1	1
Are reported indices of reliability adequate?	1	1	1	1	1	1	1	1	1	1
Construct validity										
Is there reported mathematical justification that a single scale or multiple subscales exist in the PRO measure (e.g.: factor analysis, item response theory (IRT))?	0	0	1	1	1	1	1	0	1	1
Is the PRO measure intended to measure change over time? If yes, is there evidence of both test‐retest reliability and responsiveness change? Otherwise, there may be an explicit statement that this PRO measure, is not intended to measure change over time.	1	1	1	1	1	1	1	1	1	1
Are there findings supporting expected correlations with existing PRO measures or other clinical data?	1	1	1	1	1	1	1	1	1	1
Are there findings supporting expected differences in scores between known groups?	0	0	1	0	1	1	1	1	0	1
Scoring and Interpretation										
Is there documentation how to score the PRO measure?	1	1	1	1	1	1	1	1	1	1
Has a plan for managing and/or interpreting missing responses been described?	0	0	0	0	1	1	0	0	0	0
Is there information on how to interpret the PRO measure scores?	0	1	1	1	1	1	1	1	1	1
Respondent burden and presentation										
Is time to complete reported and reasonable? If not, are number of questions appropriate for the intended application?	0	0	1	1	1	1	0	0	0	1
Is there a description of the literacy level of the PRO measure?	0	0	0	1	1	1	0	0	0	0
Is the entire PRO measure available for public viewing?	1	1	1	1	1	1	1	1	1	1

^a^ PROM`s used; #score‐0/1‐criterion not met/ criterion met.

The main properties and uses of each PROM will be presented in the following.

The Cutaneous Body Image Scale (CBIS) is a 7‐item (10 point) Likert scale from 0 ‘not at all’ to 9 ‘very markedly’. ‘I like the overall appearance of my skin’. The CBIS has been used and validated in multiple skin diseases by the construction team^15^; it has been used in patients with psoriasis, atopic dermatitis, and acne.^16^, ^17^

The Japanese version of CBIS has been used in dermatitis, acne, alopecia, psoriasis, and skin tumours.^18^

The Body Image Quality of Life Inventory (BIQLI)^19^, ^20^, ^21^ is a 19 item 7‐point Likert scale from −3 very negative to +3 very positive: ‘How confident I feel in my everyday life’. The BIQLI has been used in cutaneous lupus erythematosus^22^ in facial palsy before and after botulinum toxin injection^23^ before and after injectable procedures for facial ageing.^24^

The BIQLI has been adapted to Danish.^25^ The Danish version has been used in Hidradenitis Suppurativa.^26^

The Body Image scale (BIS)^27^, ^28^ was developed for use in cancer patients. It is a 10 item Likert scale from score 0 ‘not at all’ to score 3 ‘very much’. The BIS has been used in Cutaneous lupus erythematosus and in skin tumours.^29^

The Portuguese version of BIS has been used in skin tumours and breast cancer.^30^, ^31^, ^32^

The Appearance Schema's Inventory Revised (ASI‐R) is a 20 item, 5‐point Likert scale from 1 (strongly disagree) to 5 (strongly agree). It contains two subscales. (1) Self‐evaluative salience and (2) Motivational salience.^33^ The questionnaire has been used in psoriasis^34^, ^35^ and in pemphigus.^36^

The Body Image State Scale (BISS) is a Likert scale with six nine‐point items.^37^ Low scores reflect more negative body image. This questionnaire has been used in hyperhidrosis.^38^

The Spanish version of BISS was thoroughly validated in diverse groups and compared to other questionnaires.^39^

The Body‐Self Relations Questionnaire (BSRQ) contains 10 subscales and consists of 69 Likert type items with five grades from 1 ‘never’ to 5 ‘very often’. The questionnaire is available in a shorter version containing only five subscales with 35 items.^40^ This questionnaire has been used in psoriasis.^41^

## 4 DISCUSSION

The aim of this systematic review was to identify and evaluate the Body Image PROMS used in studies involving dermatologic conditions. In general, there is a paucity of studies investigating body image in dermatology and hence scarcer those fully evaluating the use of PROMs in this domain. A total of five PROMs were reported more than once within a 10‐year period. One instrument used only once was chosen as it was previously validated.

However, there were few descriptions of item development, and in three cases factor analysis was not performed.

An evaluation of expected differences between known groups was not undertaken in four proms. There was a lack of sensibility in everyday practical use of the PROMs regarding patient involvement in study design. A plan for missing scores were found only in two cases. Time to complete was not stated in any but had to be derived from the number of items. The literacy level of the PROM was only measured in three cases. Only one PROM (The BIS) ticked all the boxes in Table 2.

One of the main limitations in this study was the use of the COSMIN checklist. Several reviews have used the Cosmin approach^42^, ^43^ others have not.^44^, ^45^ It remains the gold standard in the assessment of PROMs.^9^ It was devised between 2006 and 2010 and consists of 119 items over 10 categories which may limit its usefulness. We therefore used the simplified approach devised by Francis.^10^ It should be noted that the relative importance of a specific measurement property may vary substantially with the purpose and context of the PROMs use. It was decided not to use a total sore as this would imply that each item should be weighted equally.^10^ It is not recommended to use The Cosmin checklist in evaluation of PROMS developed using Modern Test Theory (MTT), but only those developed using classical test theory (CTT). MTT incorporates Item response modelling, which includes a Rasch analysis. CTT is based on simple mathematics, primarily averages, proportions, and correlations. Hence COSMIN is a major limitation to further progress in the field.^46^ Psychometric criteria (COSMIN) are often inadequate in the setting of clinical assessment because of their quest for homogeneity of components and lack of attention to clinical utility and sensitivity in the real‐world environment.^47^, ^48^

This study has highlighted that patient involvement in development of PROMs should be encouraged. Use of PROMs without patient input is increasingly being viewed as unwise and perhaps unethical.^46^ Also, health outcome measures require new, better‐quality PROMs, that aim to produce theory‐based item calibration, approaching the standards of measurements found in physical science.^46^ Item calibration is part of the larger topic of item response theory (IRT). The goal of item calibration is to develop a pool or bank of items which are on the same scale.

In conclusion, this study has demonstrated the limitations in data interpretation of studies investigating the effect of body image perception in dermatologic disease and calls for further research in the field. This will rely on the development of high‐ quality validated PROMs with patient involvement which can extend to specific psychological conditions such as Body Dysmorphic Disorder. Ultimately further research will equip clinicians with a better understanding of the relationship in the context of dermatologic disease and enable timely treatment and support for this high‐risk group of patients.

## AUTHOR CONTRIBUTIONS

**Johannes Kjeldstrup Kristensen**: Conceptualization (lead); Data curation (lead); Formal analysis (lead); Funding acquisition (equal); Investigation (lead); Methodology (lead); Project administration (lead); Resources (equal); Software (equal); Supervision (lead); Validation (lead); Visualization (lead); Writing – original draft (lead); Writing – review & editing (equal). **Corina Nielsen**: Conceptualization (equal); Data curation (equal); Formal analysis (equal); Funding acquisition (equal); Investigation (equal); Methodology (equal); Project administration (supporting); Resources (supporting); Software (supporting); Supervision (supporting); Validation (supporting); Visualization (equal); Writing – original draft (supporting); Writing – review & editing (supporting). **Nora Haloob**: Conceptualization (supporting); Data curation (supporting); Formal analysis (supporting); Investigation (supporting); Methodology (equal); Project administration (equal); Resources (equal); Software (equal); Supervision (equal); Validation (supporting); Visualization (equal); Writing – original draft (supporting); Writing – review & editing (equal).

## CONFLICT OF INTEREST

None to declare.

## Data Availability

Data available openly.

## References

[1] Cash TF , Pruzinsky T , editors. Body image: a handbook of theory, research, and clinical practice. New York: Guilford Press; 2002.

[2] Didie ER , Kunuiega‐Pietzak T , Phillips KA . Body image in patients with body dysmorphic disorder: evaluations of and investment in appearance, health/illness, and fitness. Body Image. 2010;7(1):66–9. 10.1016/j.bodyim.2009.09.007 19942488

[3] Yarmohammadi S , Ghaffari M , Yarmohammadi H , Koukamari PH , Ramezankhani A . Relationship between quality of life and body image perception in Iranian medical students: structural equation modelling. Int J Prev Med. 2020;11:159–65. 33312468 10.4103/ijpvm.IJPVM_203_19 PMC7716616

[4] Cerea S , Bottesi G , Grisham JR , Ghisi M . Non‐weight related body image concern and body dysmorphic disorder prevalence in patients with anorexia nervosa. Psychiatr Res. 2018;267:120–5. 10.1016/j.psychres.2018.05.068 29886274

[5] Noles SW , Cash TF , Winstead BA . Body image, physical attractiveness and depression. J Consult Clin Psychol. 1985;53(1):88–94. 10.1037/0022-006x.53.1.88 3980834

[6] Herbst I , Jemec GBE . Body dysmorphic disorder in dermatology. Psychiatr Q. 2020;91(4):1003–10. 10.1007/s11126-020-09757-y 32472234

[7] Dalgard FJ , Gieler U , Tomas‐Aragones L , Lien L , Poot F , Jemec GBE , et al. The psychological burden of skin diseases: a cross sectional multicenter study among dermatological out‐patients in 13 European countries. J Invest Dermatol. 2015;135(4):984–91. 10.1038/jid.2014.530 25521458 PMC4378256

[8] Moher D , Shamseer L , Clarke M , Ghersi D , Liberati A , Petticrew M , et al. Preferred reporting items for systematic review and meta‐analysis protocols (PRISMA‐P) 2015 statement. Syst Rev. 2015;4:1–9. 10.1186/2046-4053-4-1 25554246 PMC4320440

[9] Terwee CB , Bot SDM , de Boer MR , van der Windt DAWM , Knol DL , Dekker J , et al. Quality criteria were proposed for measurement properties of health status questionnaires. J Clin Epidemiol. 2007;60(1):34–42. 10.1016/j.jclinepi.2006.03.012 17161752

[10] Francis DO , McPheeters ML , Noud M , Penson DF , Feurer ID . Checklist to operationalize measurement characteristics of patient‐reported outcome measures. Syst Rev. 2016;5(1):129–40. 10.1186/s13643-016-0307-4 27484996 PMC4971647

[11] Thompson AR . Body image issues in dermatology. In: Cash T and Smolak L , editors. Body image. New York: The Guilford Press; 2012, p 323‐32, 2nd. ed.

[12] Cronbach LJ . Coefficient alpha and the internal structure of tests. Psychometrika. 1951;16(3):297–334. 10.1007/bf02310555

[13] Mokkink LB , Terwee CB , Patrick DL , Alonso J , Stratford PW , Knol DL , et al. The COSMIN checklist for assessing the methodological quality of studies on measurement properties of health status measurement instruments: an international Delphi study. Qual Life Res. 2010;19(4):539–49. 10.1007/s11136-010-9606-8 20169472 PMC2852520

[14] Prinsen CAC , Mokkink LB , Bouter LM , Alonso J , Patrick DL , de Vet HCW , et al. COSMIN guidelines for systematic reviews of patient‐reported outcome measures. Qual Life Res. 2018;2(5):1147–1157. 10.1007/s11136-018-1798-3 PMC5891568 29435801

[15] Gupta MA , Gupta AK , Johnson AM . Cutaneous body image: empirical validation of a dermatologic construct. J Invest Dermatol. 2004;123(2):405–6. 10.1111/j.0022-202x.2004.23214.x 15245443

[16] Amr M , Kaliyadan F , Shams T . Use of a Cutaneous Body Image (CBI) scale to evaluate self‐perception of body image in acne vulgaris. Acta Derm Venereol. 2014;22:196–9. 25230060

[17] Hinkley SB , Holub SC , Menter A . The validity of cutaneous boy image as a construct and a mediator of the relationship between cutaneous disease and mental health. Dermatol Ther. 2020;10(1):203–11. 10.1007/s13555-020-00351-5 PMC6994570 31950338

[18] Higaki Y , Watanabe I , Masaki T , Kamo T , Kawashima M , Satoh T , et al. Japanese version of cutaneous boy image scale: translation and validation. J Dermatol. 2009;36(9):477–84. 10.1111/j.1346-8138.2009.00690.x 19712274

[19] Cash TF , Grasso K . The norms and stability of new measures of the multidimensional body image construct. Body Image. 2005;2:199–203. 10.1016/j.bodyim.2005.03.007 18089188

[20] Cash TF , Jakatdar TA , Williams EF . The body image quality of life inventory: further validation with college men and women. Body Image. 2004;1(3):279–87. 10.1016/s1740-1445(03)00023-8 18089159

[21] Cash TF , Fleming EC . The impact of body image experiences: development of the body image quality of life inventory. Int J Eat Disord. 2002;31(4):455–60. 10.1002/eat.10033 11948650

[22] Jolly M , Kazmi N , Mikolaitis RA , Sequeira W , Block JA . Validation of the cutaneous lupus disease area and severity index (CLASI) using physician‐ and patient‐assessed health outcome measures. J Am Acad Dermatol. 2013;68(4):618–23. 10.1016/j.jaad.2012.08.035 23107310

[23] De Carvalho VF , Vieira APS , Paggiaro AO , Salles AG , Gemperli R . Evaluation of body image of patients with facial palsy before and after the application of botulinum toxin. Int J Dermatol. 2019;58(10):1175–83. 10.1111/ijd.14414 30907435

[24] Sobanko JF , Dai J , Gelfand JM , Sarwer DB , Percec I . Prospective cohort study investigation changes in body image, quality of life and self‐esteem following minimally invasive cosmetic procedures. Dermatol Surg. 2018;44(8):1121–8. 10.1097/dss.0000000000001523 29659404

[25] Rasmussen TB , Berg SK , Dixon J , Moons P , Konradsen H . Instrument translation and initial psychometric evaluation of the Danish body image quality of life inventory. Scand J Caring Sci. 2016;30(4):830–44. 10.1111/scs.12311 26773708

[26] Andersen PL , Nielsen RM , Sigsgaard V , Jemec GBE , Riis PT . Body image quality of life in patients with hidradenitis suppurativa compared with other dermatological diseases. Acta Derm Venereol. 2020;100(8):adv00107. 10.2340/00015555-3464 adv 00107. 32201901 PMC9128911

[27] Hopwood P , Fletcher I , Lee A , Al Ghazal S . A body image scale for use with cancer patients. Eur J Cancer. 2001;37(2):189–97. 10.1016/s0959-8049(00)00353-1 11166145

[28] Melissant HC , Neijenhuijs KI , Jansen F , Aaronsen NK , Groenvold M , Holzner B , et al. A systematic review of the measurement properties of the body image scale (BIS) in cancer patients. Support Care Cancer. 2018;26(6):1715–26. 10.1007/s00520-018-4145-x 29532245 PMC5919987

[29] Ogunsanya ME , Cho SK , Hudson A , Chong BF . Factors associated with quality of life in cutaneous lupus erythematosus using the revised Wilson and Cleary Model. Lupus. 2020;29(13):1691–703. 10.1177/0961203320951842 32883161 PMC7641991

[30] Moreira H , Silva S , Marques A , Canavarro MC . The Portuguese version of the body image scale (BIS) – psychometric properties in a sample of breast cancer patients. Eur J Oncol Nurs. 2010;14(2):111–18. 10.1016/j.ejon.2009.09.007 19892597

[31] Pereira MG , Ponte M , Ferreira G , Machadp JC . Quality of life in patients with skin tumors: the mediator role of body image. Psycho Oncol. 2017;26(6):815–21. 10.1002/pon.4236 27502437

[32] Pereira MG , Baia V , Machado JC . Coping and quality of life in patients with skin tumors in the follow up stage: the mediating role of body image and psychological morbidity. J Psychosoc Oncol. 2016;34(5):400–12. 10.1080/07347332.2016.1196807 27564996

[33] Cash TF , Melnyk SE , Hrabosky JI . The assessment of body image investment: an extensive revision of the appearance schemas inventory. Int J Eat Disord. 2004;35(3):305–16. 10.1002/eat.10264 15048946

[34] Wojtyna E , Lakuta P , Marcinkiewicz K , Bergler‐Czop B , Brzezinska‐Wcislo L . Gender, body image and social support: biopsychosocial determinants of depression among patients with psoriasis. Acta Derm Venerol. 2017;97(1):91–7. 10.2340/00015555-2483 27304233

[35] Lakuta P , Marcinkiewicz K , Bergler‐Czop B , Brzezinska‐Wcisio L . The relationship between psoriasis and depression: a multiple mediation model. Body Image. 2016;19:126–32. 10.1016/j.bodyim.2016.08.004 27690315

[36] Mazotti E , Mazotti A , Antinone V , Alfani S , Cianchini G , Abeni D . Psychological distress, and investment in one’s appearance in patients with pemphigus. J Eur Acad Dermatol Venereol. 2011;25(3):285–9. 10.1111/j.1468-3083.2010.03780.x 20626535

[37] Cash TF , Fleming EC , Alindogan J , Steadman L , Whitehead A . Beyond body image as a trait. The development and validation of the body image states scale. Eat Disord. 2002;10(2):103–13. 10.1080/10640260290081678 16864251

[38] Kristensen JK , Möller S , Vestergaard DG , Horsten H‐H , Swartling C , Bygum A . Anxiety and depression in primary hyperhidrosis: an observational study of 95 consecutive Swedish outpatients. Acta Derm Venereol. 2020;100(15):adv00240. 10.2340/00015555-3598 32725249 PMC9207642

[39] Chams MM , Tinico L , Mejia‐Rodriguez D , Martinez‐Banfi ML , Preuss H , Hammerie F , et al. The Spanish body image states scale: factor structure, reliability, and validity in a Columbian population. Front Psychol. 2019;10:2553. 10.3389/fpsyg.2019.02553 31824372 PMC6883919

[40] Brown TA , Cash TF , Mikulka PJ . Attitudinal body image assessment: factor analysis of the body‐self relations questionnaire. J Pers Assess. 1990;55:135–44. 2231236 10.1080/00223891.1990.9674053

[41] Rosinska M , Rzepa T , Szramka‐Pawlak B , Zaba R . Body image and depressive symptoms in persons suffering from psoriasis. Psychiatr Pol. 2017;51(6):1145–52. 10.12740/pp/68948 29432509

[42] Heinl D , Prinsen CAC , Deckert S , Chalmers JR , Drucker AM , Ofenloch R , et al. Measurement properties of adult quality‐of‐life measurement instruments for eczema: a systematic review. Allergy. 2016;71(3):358–70. 10.1111/all.12806 26564008

[43] Sundaram CS , Dhillon HM , Butow PN , Sundaresan P , Rutherford C . A systematic review of body image measures for people diagnosed with head and neck cancer. Support Care Cancer. 2019;27:3657–66. 31203508 10.1007/s00520-019-04919-6

[44] Noud M , Hovis K , Gelbard A , Sathe NA , Penson DF , Feurer ID , et al. Patient reported outcome measures in upper airway‐related dyspnoe. A systematic review. Otolaryngol Head Neck Surg. 2017;143(8):824–31. 10.1001/jamaoto.2017.0348 PMC5604091 28594976

[45] Alrubaiy L , Hutchings HA , Williams JG . Assessing patient reported outcome measures: a practical guideline for gastroenterologists. United Eur Gastroenterol J. 2014;2(6):463–70. 10.1177/2050640614558345 PMC4245310 25452841

[46] McKenna P , Heany A . Setting and maintaining standards for patient‐reported outcome measures: can we rely on the COSMchecklist. J Med Econ. 2021;24:502–11. 33759686 10.1080/13696998.2021.1907092

[47] Carrozzino D , Patierno C , Guidi J , Montiel CB , Cao J , Charlson M , et al. Clinimetric criteria for patient reported outcome measures. Psychother Psychosom. 2021;90(4):222–32. 10.1159/000516599 34038901

[48] Bech P . Clinical psychometrics. Oxford: Wiley‐Blackwell; 2012. p. 200.

